# Prevalence, Pattern and Predictors of Mental Health Morbidity Following an Intermediate Disaster in an Urban Slum in Delhi : A Modified Cohort Study

**Published:** 2004

**Authors:** Nimesh G Desai, Dhanesh K Gupta, Raj Kumar Srivastava

**Affiliations:** 1 Prof. & Head, Dept. of Psychiatry and Medical Superintendent, Institute of Human Behaviour and Allied Sciences (IHBAS), Dilshad Garden, Delhi 110 095 Phone: 22113395 Fax: 22599227 ngd2000@hotmail.com; 2 Associate Professor, Dept. of Psychiatry, Institute of Human Behaviour and Allied Sciences, Dilshad Garden, Delhi 110 095, India; 3 Senior Resident, Dept. of Psychiatry, Institute of Human Behaviour and Allied Sciences, Dilshad Garden, Delhi 110 095, India

**Keywords:** Disaster, Mental Health, Prevalence, Pattern, Predictors

## Abstract

The present study reports on the findings from an ICMR supported Research Project on the mental health consequences and service needs of the population of an urban slum in Delhi affected by an intermediate fire disaster. The study was aimed at examining the prevalence, the pattern and the predictors of mental health morbidity in the disaster affected population. Modified cohort design was used , with a control group, and two stage assessments for the prevalence of psychiatric disorder at two years after the disaster, with GHQ-12 and SCAN based clinical interview with ICD-10-DCR.. The data were analysed using r2 test and independent ′t′ test for inter group comparison and stepwise logistic regression for finding predictors of psychiatric morbidity and psychological ill health. The prevalence of psychiatric disorders was significantly higher (78/1,000 v/s 22/1,000), and the prevalence of psychological ill health was also higher (232/1000 v/s 50/1000), as compared to the control group. The commonest psychiatric disorders were Depression, Substance Use Disorders, Generalised Anxiety Disorder, and Somatoform Disorders. The commonest symptoms of psychological ill health were suggestive of depression. Age and participation in relief work were found to be strong predictors, and physical injuries were found to be a weak predictor of mental health morbidity. The findings have important implications in the service delivery and research on mental health aspects of disasters, which are highlighted and discussed.


*Indian Journal of Psychiatry, 2004, 46(I)39-51*

### Prevalence, Pattern and Predictors of Mental Health Morbidity following an Intermediate Disaster in an Urban Slum in Delhi: A Modified Cohort Study

**Nimesh G. Desai*[1], Dhanesh K. Gupta[2], Raj Kumar Srivastava[3]**

**ABSTRACT**

The present study reports on the findings from an ICMR supported Research Project on the mental health consequences and service needs of the population of an urban slum in Delhi affected by an intermediate fire disaster. The study was aimed at examining the prevalence, the pattern and the predictors of mental health morbidity in the disaster affected population. Modified cohort design was used , with a control group, and two stage assessments for the prevalence of psychiatric disorder at two years after the disaster, with GHQ-12 and SCAN based clinical interview with ICD-10-DCR.. The data were analysed using $r^2$ test and independent 't' test for inter group comparison and stepwise logistic regression for finding predictors of psychiatric morbidity and psychological ill health. The prevalence of psychiatric disorders was significantly higher (78/1,000 v/s 22/1,000), and the prevalence of psychological ill health was also higher (232/1000 v/s 50/1000), as compared to the control group. The commonest psychiatric disorders were Depression, Substance Use Disorders, Generalised Anxiety Disorder, and Somatoform Disorders. The commonest symptoms of psychological ill health were suggestive of depression. Age and participation in relief work were found to be strong predictors, and physical injuries were found to be a weak predictor of mental health morbidity. The findings have important implications in the service delivery and research on mental health aspects of disasters, which are highlighted and discussed.

Key Words: Disaster, Mental Health, Prevalence, Pattern, Predictors

### INTRODUCTION

A disaster is a severe disruption - ecological and psychological, which greatly exceeds the coping capacity of the affected community (WHO, 1992a). Disasters traumatically expose normal populations to severe threats to life, death of people and massive environmental destruction. It has been known that disasters place the affected individuals under tremendous pressure to cope and adjust under physically and psychologically hostile conditions. In the later part of the 20th century mental health scientists and social scientists started investigating the psychosocial consequences of disaster. There is paucity of research on the psychosocial effects of disasters in the developing countries. In India, researchers and mental health teams have been studying psychiatric morbidity in the communities affected by disasters like flood, drought, cyclone, earthquake, refugee situations, starting with the Bhopal gas tragedy in 1984. The National Workshop on Psychosocial Consequences of Disasters organized in 1997 has recommended that disaster research related to mental health aspects should include all kinds of disasters (NIMHANS, 1997)

Disasters have been classified in many ways like based on their aetiology, impact and the required response. In terms of the impact and the required response, disasters have been classified as central, intermediate and peripheral (Green, 1982).

- CENTRAL TYPE OF DISASTERS - Disasters in which the whole physical and organizational structure of the community is deeply changed (e.g. earthquake, flood, cyclone, etc.) because homes are destroyed. Such disasters are seen as major events, elicit considerable response and are often the target of attention. The Bhopal gas tragedy, is a well understood example of this type. Mental health research on some disasters of this type, has been carried out in India by various teams starting from Bhopal gas tragedy and carrying on to the Latur earthquake, Orissa cyclone and Gujarat earthquake.
- INTERMEDIATE TYPE OF DISASTERS - Intermediate disasters are events which affect a group of people within a community in some areas, but there are still unaffected members of the community and the

[1]Prof. & Head, Dept. of Psychiatry and Medical Superintendent, Institute of Human Behaviour and Allied Sciences (IHBAS), Dilshad Garden, Delhi 110 095 Phone: 22113395 Fax: 22599227 E-mail: ngd2000@hotmail.com, ngd2000@rediffmail.com

[2]Associate Professor, Dept. of Psychiatry, Institute of Human Behaviour and Allied Sciences, Dilshad Garden, Delhi 110 095

[3]Senior Resident, Dept. of Psychiatry, Institute of Human Behaviour and Allied Sciences, Dilshad Garden, Delhi 110 095

*Author for Correspondence physical settings (homes, neighbourhood) remain unchanged, and people are not displaced (e.g., fire in an residential colony). Disasters of this type are more frequent, specially in the urban area in the current time. They have received some attention and usually elicit modest response. Fire in residential colonies, home collapse, land slides and nuclear disasters are some examples of this type. There has generally not been adequate attention given to the mental health research in this type of disasters in India, except for some work carried out in Bombay following the communal riots of 1992.

- PERIPHERAL TYPE OF DISASTERS - Disaster happens to a group of people who come together by chance (e.g. an aeroplane crash or hijack). Survivors return to their respective geographic communities where the physical settings and social support networks are still intact.

The present study was carried out as a consequence of a service delivery initiated at the time of a fire in an urban slum in Delhi, which can be classified as an intermediate disaster. At the time of the fire, which created significant loss of property and physical injuries, along with some loss of life, a mental health team joined the other agencies in the field for participating in the relief work. As a consequence of this initiative and in view of the need for systematic research on the epidemiology of mental health morbidity following disasters, particularly of the intermediate type, a research proposal was developed. In the event, the time required for the development and formulation of the research proposal was long, followed by the review by the funding agency i.e. the Indian Council of Medical Research (ICMR). As such, the focus of research was on long term mental health morbidity in a representative sample, with a control group. This paper describes the finding of the ICMR research project, on the prevalence, the pattern and the predictors of mental health morbidity in the disaster affected population (Gupta & Desai, 2003).

### REVIEW OF LITERATURE

As described earlier, the mental health and social science interest in the psychosocial effects of disasters has received attention in the later part of the 20th century. The available evidence has been reviewed and recommendations have been made by the World Health Organization in 1992 and at a National Workshop in India in 1997. The large amount of international research information has also been analysed and reviewed by Rubonis and Bickman (1991) and Bromet and Dew (1995). There has been considerable work on the mental health aspects of disasters in India in the past two decades, in terms of service delivery, training of professionals and some systematic research. Disasters occur both in developed as well as developing countries probably in the same frequency (Berz, 1989). However, the impact of disaster is more severe and effects more devastating in the developing countries (WHO, 1992a).

### INTERNATIONAL STUDIES ABOUT MENTAL HEALTH CONSEQUENCES OF DISASTERS:

Rubonis & Bickman (1991) reviewed and analysed the relationship between disasters and subsequent psychopathology in 52 studies, which used quantitative measures. The authors examined relationship among four sets of variables related to disaster and subsequent psychopathology. The characteristics of the victim population, characteristics of the disaster, the study methodology, and the type of psychopathology.

In the studies examined, between 7% and 40% of all subjects showed some form of psychopathology. The type of psychopathology with the highest prevalence rate was general anxiety (40%). Phobic symptoms (32%), psychosomatic symptoms (36%), alcohol abuse (36%), appeared to show slightly lower level of prevalence, with depression (26%) and drug abuse (23%) somewhat lower still.

Using meta-analysis technique the authors showed that in these studies a positive relationship emerged between disaster occurrence and psychopathology, indicating an increase of 17% in the prevalence rate of psychopathology (compared with a pre-disaster or control group rate) as a result of a disaster. The number of female victims in the sample studied, the death rates and the amount of time that had lapsed since the disaster event were all directly related to the amount of psychopathology.

In another review of 24 studies, it has been found that in the studies published since meta-analysis by Rubonis and Bickman (1991) there continues to be a moderate, statistically significant excess in morbidity associated with exposure. In addition, advances in delineation of PTSD have led to its inclusion in several recent studies (Bromet and Dew, 1995).

### INDIAN STUDIES

Mental health experts have vast experience of handling the disasters in India during last two decades. Although this experience is mainly limited to service delivery and training, there have been well planned research studies also.

The Bhopal gas tragedy (1984), Marathawada earthquake (1993), Andhra Pradesh Cyclone (1996) and Jabalpur earthquake (1997), the Orissa cyclone (1999) and the

Gujarat earthquake (2001) have been the central type of disasters in which mental health professionals have taken an active part in terms of providing mental health services and undertaking research to study the psychosocial impact of these disasters. Sethi et al (1987) in a study of out patients attending 10 Government clinics located in the gas exposed areas found that 22.6% of the adult outpatients were suffering from mental disorder and this figure was reported to be higher than that of observed in a general health service settings using a comparable methodology. In a community based study on representative sample of gas exposed population with a control group of unexposed population, the prevalence of psychiatric morbidity in exposed population after one and a half year of disaster was found to be significantly higher in comparison to control group (94 per thousand v/s 25 per thousand) (Bhiman, 2001). Further the prevalence of psychiatric morbidity decreased to 48 per thousand by 5th year post disaster (Bhiman, 2001).

A study by Sharan et al (1996) from Latur earthquake in Marathwada on a sample of 56 adults in rural area after a month of disaster showed a psychiatric morbidity of 59% among the affected population of which PTSD (23%) and major Depression (21%) were the most common diagnosis. In a study on the same earthquake. The ICMR carried out an extensive longitudinal study in Latur for determining the nature and prevalence of psychiatric morbidity and documentation of physical health complaints and vital statistics. The study also assessed various dimensions of exposure to the disaster and subsequent stresses and mediating factors in the form of adverse life events and situations, social support etc. The study found that 21.46% adult males in the affected group received a psychiatric diagnosis compared to 13.14% in the control group. The corresponding figures for adult females were 14.99% and 5.05% respectively. The disaster affected subjects reported less number of desirable and more undesirable life events than control group. Significantly higher proportion of respondents in the affected sample reported dissatisfaction with social support while as those reporting 'feeling very satisfied' were also more in the same group (ICMR, 2000).

The pilot phase of an ICMR project on mental health service needs and service delivery models in earthquake affected areas of Gujarat, carried out during the first year after the disaster (Desai et al, 2002) made a broad based assessment of mental health aspects of earthquake disaster in Gujarat by examining the whole phenomenon with normative approach. It was concluded that there was evidence for definitive need to focus on the emotional and psychological needs of the population in dealing with the post disaster situation, but simultaneously there was need to be cautious not to over psychiatricise the issue. A three level model of psychological disturbances was suggested about the mental health consequences and the required response (Desai et al, 2002).

(i) Mild to moderate psychological transient disturbance of emotion and/or thoughts which occur in a very large population (70-90% of the population)

(ii) Moderate to severe psychological disturbances, subsyndromal psychiatric problems and acute stress related disorders (30 to 50% of population)

(iii) Diagnosable psychiatric disorders mostly related to stress, which may begin to occur any time after 2-3 months of the disaster and will require specialized mental health services (5-15% of population).

There are some reports of psychiatric morbidity following peripheral disasters, which are obviously seen in the victims and their families. The first report by Narayanan et al (1987) had reported on the grief reaction among 137 bereaved relatives of 70 victims of a fire disaster in a circus in Bangalore city in 1981. They found 49 (35.76%) bereaved relatives with symptoms requiring treatment. Gautam et al (1998) reported prevalence of psychiatric morbidity of 35.45% within days of a bomb blast. Sharma et al (1998) found a high rate of 56% of Post Traumatic Stress Disorder (PTSD) in the children affected by a fire disaster.

Little information is available on the impact of intermediate disasters. The communal riots of Bombay, a report on which is available (Shetty and Chhabria, 1997), although intermediate in type, had significant impact on psychological and social issues of the entire city. As such it may well be classified as a central disaster. There is no other systematic research information on mental health morbidity following intermediate disasters.

### BACKGROUND OF INDEX DISASTER AND INITIATION OF THE RESEARCH PROJECT

On March 14, 1999, a devastating fire broke out in the urban slum area of Yamuna Pushta of Delhi. This was one of the worst fire tragedies in the recent time wherein at least 32 people were killed and a large number injured in stampede due to panic. A majority of the affected families lost their entire personal belongings including their already inadequate property. A few families sustained major setback where only a single member survived while the remaining perished in the fire.

On March 15, a field team of 15 persons, comprising of psychiatrists, psychologists and social workers was constituted by IHBAS with the initial objective of providing mental health services, as part of the health and general relief service by the other agencies. The experience in the initial works was mixed along with the community acceptance of the services, although the local health providers and the community leaders were very keen on the mental health service component.

A research proposal was developed and submitted to ICMR for funding in July 1999, with the twin purpose of specifically addressing research issues and continuity of the services in the affected colony. In the period from July 1999 to Dec. 2000 when the funded project was initiated for studying a long term mental health morbidity, the participation in the relief work and other community activities continued by the regular cadre staff and student volunteers.

### Rationale for the study

It can be summarized from the review of literature above that a large part of the research on the epidemiology of mental health morbidity following disasters has been in situations of central disaster with a cross section of the general population. In the Indian studies of this nature, the prevalence rate for psychiatric morbidity has ranged from 9.4% to 59%. None of these studies have reported on sub-syndromal symptoms or ill-health.

On the other hand there have been studies on the psychiatric morbidity in peripheral disaster situations. In these situations per se, the target population is of the victims / survivors and their families. There is no community which is affected and no target general population to be studied. Indian studies of this nature have reported prevalence rate from 35% to 66%.

The intermediate disaster situation of the kind studied here, has very high impact on the affected general population of a certain geographical area, along with victims and survivors. The fire disaster in the Yamuna Pushta Colony affected the general population of an urban slum which is socio economically disadvantaged and underprivileged. There have been no epidemiological studies with focus on psychiatric disorders and psychological ill-health in a disaster affected population which is socio economically underprivileged. The present study is the first study of this kind in an intermediate disaster situation, with implications for the impact of disasters in general, and of intermediate disaster in particular, specifically in underprivileged communities of urban slum.

### RESEARCH QUESTIONS

1. What is the prevalence of mental health morbidity (psychiatric disorders and psychological ill-health) in disaster affected populations, and is it different from control population?
2. What is the pattern of mental health morbidity (psychiatric disorder and psychological ill-health) in disaster affected populations?
3. What are the predictors of mental health morbidity (psychiatric disorders and psychological ill health) in disaster affected populations?

### HYPOTHESES

H0: There will be no difference in mental health morbidity in disaster affected population and control population.

H1: The prevalence of mental health morbidity will be higher in disaster affected population than control population.

### OBJECTIVES

### General Objective

To study the epidemiology of mental health morbidity in the population of an urban slum affected by a major fire disaster of intermediate type.

### Specific Objectives

1. To study the prevalence rate of psychiatric disorders and psychological ill health in disaster affected population, in comparison to the control population.
2. To study the pattern of psychiatric disorders and psychological ill health in disaster affected population.
3. To identify the predictor variables for psychiatric disorders and psychological ill health.

### METHODOLOGY

a) **Universe of study -** Individuals in the intermediate disaster affected communities in the urban slums in India.

b) **Study Design -** The study employed a modified cohort design in which the cohort comprised of a study group sample from disaster (fire) affected area (exposed) and an independent control group sample from a non-affected area (unexposed). The individuals were selected after the exposure occurred. This has been accepted as a design for modified cohort study in disaster situations (Bromet and Dew, 1995).

c) **Sample**

(i) Sampling unit - Household was taken as the sampling unit.

(ii) Study unit - Individual members of the households fulfilling the study criteria were enrolled as study subjects. All persons aged 16 years or above were included in the study. In addition, in case of study group, the individual must have been residing in the area at the time of fire tragedy. Those who refused consent or could not be contacted on at least three visits to their houses were excluded.

(iii) Sample size - sample size for a confidence level of 95% and power of 80% was calculated by taking expected prevalence of psychiatric morbidity in the non affected (control) group as 8% based on the prevalence of psychiatric morbidity in urban population in India (Reddy & Chandrasekhar, 1998).

(iv) Sample selection - the sample comprised of a study group from the disaster (fire) affected Bengali colony in Yamuna Pushta slum cluster in Delhi and an independent control group from Hathibasti, a non affected colony in the same slum cluster. The choice of colony for control group was based on the criteria that

- There was no exposure to the effect of fire disaster, as it is located 4 km away from the fire affected colony (Bengali Colony).
- There has not been any fire tragedy or other major calamity in this colony during past 10 years
- Socio economically, it seemed to be comparable with the fire-affected colony.

All the hutments in both the colonies were enumerated with the help of key persons in the colonies to prepare the sampling frame. A sample of 500 households was selected with the help of random number table in each colony. All the members of selected households fulfilling study criteria constituted the sample. The number of families selected were decided on the basis of estimated household size of 2-3 adults per family to get a sample of at least 1200 subjects, each in study group and control group. Toward the end of the data collection, a minor fire broke up in Hathi-Basti in which twenty households selected initially for control groups were affected. As these households were still not covered in data collection, these were excluded from control group, which finally though comprised of 480 households could provide adequate sample size.

**(d) Study Tools:**

1. Semi-structured interview schedule (SSI) - A semi-structured interview schedule specifically prepared for the study was used to collect information on sociodemographic variables in both groups and the extent of exposure to fire disaster in study group.

2. General Health Questionnaire (GHQ-12 Hindi version) - Short version (12 items) of GHQ was used for screening of subjects who are likely to have psychiatric morbidity. Those who score 2 or more were investigated further. It was also used to find out the extent and pattern of mental illhealth. The Hindi and Kannada translation of scale has been validated for use in India (Shamsunder et al, 1986; Gautam et al, 1987). The instrument was administered by the trained field research staff.

3. Schedule for Clinical Assessment in Neuropsychiatry (SCAN) (WHO, 1992b) - SCAN based interview was done by a trained psychiatrist on subjects who were screened positive in GHQ-12 to arrive at diagnosis on ICD-10 Diagnostic Criteria for Research (WHO, 1993).

**(e) Field Work Procedure**

(i) Informed Consent – The key persons with whom investigators had been in contact since the time of tragedy were appraised of the purpose of the study and their help and cooperation was taken in contacting families. Informed consent was obtained from the head of the each household included in the study. In addition, the informed consent was also obtained from each member of the household included in the study.

(ii) Assessment – Two-stage method of assessment was employed. Stage I assessment comprised of collecting, about sociodemographic information, severity and extent of exposure to the fire disaster and administration of GHQ (12 item Hindi Version). Subjects scoring equal to or more than 2 on GHQ were included for stage II assessment. In stage II assessment, the psychiatrist conducted SCAN based interview to arrive at diagnosis on ICD-10-D CR.

**(f) Data analysis -**

1. Data entry was done in EXCEL and the Data was analyzed using SPSS (8.0 version) statistical Software.

2. Prevalence of psychiatric disorders and patterns of GHQ score in both the groups were computed by using descriptive statistics

3. Groups were compared for the background variables like sociodemographic variables and dependent variables like psychiatric morbidity, GHQ score using Chi square for categorical variables and independent t-test for continuous variables was done.
4. Univariate analysis and multivariate analysis (stepwise logistic regression) were carried out for the study group with presence of psychiatric disorder as dependent variable and sociodemographic and fire exposure variables as independent variables. The similar analysis was also done with GHQ score of 2 or more as dependent variable.

### RESULTS

### Sample Characteristics

The number of subjects fulfilling the selection criteria was 1276 and 1284 in study group and control group respectively. In the study group, 1251 subjects and in the control group, 1265 subjects were included, as the remaining subjects (1.96% & 1.48%) could not be contacted despite a minimum of three visits to their houses.

**Table 1**

**Sample Selection and Attrition in the target sample**

| | Study Group | Control Group |
|---|---|---|
| Total No. of sampling units (households) | 500 | 480 |
| Total no. of units of study (eligible individuals fulfilling study criteria) | 1276 | 1284 |
| Total no. of individuals who could be included in study (GHQ) administered) | 1251 (98.04%) | 1265 (98.52%) |
| ***Individuals who could not be included**** | 25 (1.96%) | 19 (1.48%) |
| No. of subjects screened positive on GHQ | 290 | 63 |
| No. of subjects assessed by clinical interview | 256 (88.28%) | 56 (88.89%) |
| ***Individuals who could not be interviewed**** | 34 (11.73%) | 7 (11.11%) |

* Attrition

The subjects in study group were predominantly married, illiterate muslims. About half of them were unskilled or skilled workers and two third of all subjects were employed (Table 2 a & 2 b).

**Table 2 (a)**

**DESCRIPTION OF THE SAMPLE**

| Characteristic | Study Group (n=1251) | Control Group (n=1265) |
|---|---|---|
| 1. Mean Age in years±standard deviation | 32.20±12.25 | 32.11±12.74 |
| 2. Sex - | | |
| Male | 656 (52.4%) | 689 (54.5%) |
| Female | 595 (47.6%) | 576 (45.6%) |
| 3. Religion - | | |
| Hindu | 52 (4.2%) | 731 (57.8%) |
| Muslim | 1199 (95.8%) | 534 (42.2%) |
| 4. Marital Status - | | |
| Married | 1105 (88.3%) | 1004 (79.4%) |
| Single | 146 (11.7%) | 261 (20.6%) |
| 5. Per capita monthly income (Rs)±Standard Deviation | 909.01±552.31 | 646.31±349.90 |

The study group was matched with the control group for age and sex. However study group has higher per capita income, has predominantly muslim individuals and more illiterate subjects and employed subjects in comparison to the control group.

**Table 2(b)**

**DESCRIPTION OF THE SAMPLE**

| | Characteristic | Study Group (n=1251) | Control Group (n=1265) |
|---|---|---|---|
| 1. | **Education** | | |
| | ◆ No education | 1033 (82.6%) | 886 (70.1%) |
| | ◆ Upto Primary | 123 (9.8%) | 213 (16.8%) |
| | ◆ Middle | 55 (4.4%) | 99 (7.8%) |
| | ◆ Inter mediate | 27 (2.2%) | 57 (4.5%) |
| | ◆ Graduation and above | 13 (1.0%) | 10 (0.8%) |
| 2. | **Occupation** | | |
| | ◆ Unskilled/ semiskilled workers | 46 (52.4%) | 461 (36.4%) |
| | ◆ Skilled workers | 24 (1.9%) | 17 (1.3%) |
| | ◆ Businessman | 144 (11.7%) | 162 (12.8%) |
| | ◆ Service | 13 (1.1%) | 17 (1.3%) |
| | ◆ Professional | 11 (0.9%) | 1 (0.1%) |
| | ◆ Farmer | 0 (0%) | 93 (7.4%) |
| | ◆ House wife | 345 (28.0) | 362 (28.6%) |
| | ◆ Student | 0 (0%) | 1 (0.1%) |
| | ◆ None | 50 (4.0%) | 151 (12.0%) |
| 3. | **Employment Status** | | |
| | ◆ Regular employment | 792 (63.6%) | 738 (58.3%) |
| | ◆ Irregular employment | 48 (3.8%) | 13 (1.0%) |
| | ◆ Unemployed | 411 (32.6%) | 514 (40.7%) |

### Physical Impact of the Disaster

Information on the physical impact of the disaster (for study group) is provided in Table 3. A majority of the people reported complete burning of their huts and belongings. Burn injuries were sustained by 42 (3.4%) persons and non-burn physical injuries due to stampede was sustained by 32 (2.5%). Fifteen (1.2%) individuals reported death of one or more family members in the fire disaster. Individuals reporting to have participated in rescue work were 110 (8.8%) and in relief work were 320 (25.6%). As such, there had been very high impact of the disaster on the general population and sizeable population had participated in the relief work.

**Table 3**

**Physical Impact of fire disaster on study group (n=1251)**

| Physical Impact | Number of subjects (%) |
|---|---|
| 1. Destruction of Hut / House by fire | |
| ◆ Complete | 1143 (91.4%) |
| ◆ Partial | 57 (4.6%) |
| ◆ No damage | 51(4.1%) |
| 2. Destruction of Belongings | |
| ◆ Complete | 1138(91.0%) |
| ◆ Partial | 72 (5.7%) |
| ◆ No damage | 41 (3.3%) |
| 3. Burn Injury to self | |
| ◆ None | 1209 (96.6%) |
| ◆ Mild | 24 (1.9%) |
| ◆ Moderate | 2 (0.2%) |
| ◆ Severe | 16 (1.3%) |
| 4. Other physical injuries to self | |
| ◆ None | 1219 (97.4%) |
| ◆ Mild | 24 (1.9%) |
| ◆ Moderate | 3 (0.2%) |
| ◆ Severe | 5 (0.4%) |
| 5. Death of family members | |
| ◆ Yes | 15 (1.2%) |
| ◆ No | 1236 (98.8%) |
| 6. Participation in Rescue work | |
| ◆ Yes | 110 (8.8%) |
| ◆ No | 1141(91.2%) |
| 7. Participation in Relief Work | |
| ◆ Yes | 320 (25.6%) |
| ◆ No | 931 (74.4%) |

### GHQ Score

Table 4 (a) shows that mean GHQ-12 score of study group was significantly higher than that of control group (p<.001). The number of individuals showing positive score on GHQ-12 (> 2) was also significantly higher in study group (p<001) as shown in table 4(b).

**Table 4 (a)**

**GHQ Scores of Study Group and Control Group**

| | GHQ score | | t value | Sig |
|---|---|---|---|---|
| | Mean | Standard deviation | | |
| Study Group n=1251 | 1.27 | 2.34 | 13.977 | P ≤ .001 |
| Control Group n=1265 | 0.28 | 0.94 | | |

**Table 4 (b)**

**GHQ Score of Study Group and Control Group**

| GHQ score | Study Group (n=1251) | Control Group (n=1265) |
|---|---|---|
| Positive | 290 (23.2%) | 63 (5.0%) |
| Negative | 961 (76.8%) | 1202 (95.0%) |
| Total | 1251 | 1265 |

$c^2$ (df = 1) = 172.75 p≤.001

### Mental Health Morbidity

(i) Psychiatric disorder: The prevalence of psychiatric disorders in the study group was 78.8 per thousand and in control group 18 per thousand. The prevalence in the study group was significantly higher as compared to the control group (p≤.001) (Table 5). Table 6 shows the pattern of psychiatric disorders in the study group. Depressive disorders, substance use disorders and generalized anxiety disorder were the most common disorders.

**Table 5**

**Psychiatric morbidity in study group and control group**

| | Study Group (n=1217*) | Control Group (n=1260*) |
|---|---|---|
| Psychiatric disorders | | |
| Present | 96 (7.88%)** | 23 (1.83%)** |
| Absent | 1121 (92.12%) | 237 (98.17%) |
| Total | 1217 | 1260 |

$c^2$ (df = 1) = 42.437 p≤.001

* Status about presence or absence of psychiatric diagnoses was not known for 34 subjects in control group and 5 subjects in study group as clinical interview could not take place in these subjects. Hence denominator for calculating the prevalence is 1217 for the study group and 1260 for the control group.

** The prevalence rate of psychiatric disorders in study group 78.8 per thousand and in control group 18.3 per thousand.

**Table 6:**

**PATTERN OF PSYCHIATRIC DISORDERS**

| Psychiatric Disorder | No. of patients (%) | |
|---|---|---|
| | Study group (n=96) | Control group (n=23) |
| Depressive episode | 42 (43.75%) | 12 (52.17%) |
| Dysthymia | 22 (22.91%) | 4 (17.30%) |
| Disorders due to psycho-active substances | 10 (10.43%) | 1 (4.34%) |
| Generalized anxiety disorders | 5 (5.22%) | 3 (13.04%) |
| Somatoform disorders | 4 (4.16%) | 0 (0%) |
| PTSD | 0 (0%) | 1 (4.34%) |
| Others (psychosis, mania, mixed anxiety depressive disorder, adjustment disorder, etc.) | 13 (13.54%) | 2 (8.68%) |

(ii) Psychological Ill Health: Table 7 shows the item wise score on GHQ-12 for those individuals which were screened positive on GHQ-12 but did not have diagnosable syndromal disorder on SCAN based interview, and can be said to have substantial psychological ill health or symptoms not amounting to disorder. The common symptom reported by study group subjects were feeling strained, loss of sleep, not enjoying life, feeling unhappy and inability to concentrate.

**Table 7:**

**Frequency of psychological symptoms on GHQ-12 (excluding subjects with diagnosed psychiatric disorders)**

| GHQ -12 Item No. | Description of the items (Jacob & Bhugra, 1999) | No. of subjects* (%) | |
|---|---|---|---|
| | | Study group (n=194) | Control group (n=40) |
| 1. | Could not concentrate | 59 (30.41%) | 16 (40.00%) |
| 2. | Lost sleep | 72 (37.11%) | 22 (55.00%) |
| 3. | Not playing a useful part | 30 (15.46%) | 2 (5.00%) |
| 4. | Could not take decision | 22 (11.34%) | 2 (5.00%) |
| 5. | Felt under strain | 95 (48.96%) | 20 (50.00%) |
| 6. | Could not overcome difficulties | 21 (10.82%) | 11 (27.50%) |
| 7. | Not enjoying | 60 (30.92%) | 19 (47.50%) |
| 8. | Could not face problems | 34 (17.52%) | 3 (7.50%) |
| 9. | Depressed and unhappy | 58 (29.89%) | 14 (35.0%) |
| 10. | Lost confidence | 28 (14.43%) | 1 (2.50%) |
| 11. | Felt worthless | 26 (13.40%) | 0 (0%) |
| 12. | Not feeling happy | 54 (27.83%) | 11 (27.50%) |

*The total of frequencies will be above 100% as each subject has positive response on more than 1 item of GHQ

### Predictor variables of mental health morbidity:

(i) For Psychiatric Disorders – On multiple logistic regression analysis, two of the fifteen independent variables, were found to have significant strength of prediction for the dependent variable in psychiatric disorder. Higher age ($p<0.01$) and participation in relief work ($p<.001$) were found to be predictor of psychiatric disorder (Table 8). Since the age range of subjects was from 16-100 years, on further analysis, it was examined if this finding held true for the age group under 60 years of age and it was found that even after excluding the subjects with age over 60 years, the finding remained the same.

**Table 8:**

**Multiple regression analysis for psychiatric disorder as dependent variable**

| Sl. No. | Predictor Variable | OR | 95% CI | Significance |
|---|---|---|---|---|
| 1. | Age | 2.07 | 1.19-3.60 | p<.01 |
| 2. | Participation in relief work | 2.00 | 1.29-3.09 | p<.001 |

OR – Odds ratio

**Table 9:**

**Multiple logistic regression analysis for positive GHQ scores as dependent variable**

| Sl. No. | Predictor Variable | OR | 95% CI | Significance |
|---|---|---|---|---|
| 1. | Age | 2.32 | 1.61-3.36 | p<.001 |
| 2. | Participation in relief work | 1.50 | 1.12-2.01 | p<.005 |
| 3. | Other physical injuries | 5.22 | 1.21-22.43 | p<.05 |

OR – Odds ratio

(ii) For Psychological Ill health- On multiple logistic regression analysis for GHQ positive score as the dependent variable, three of the fifteen independent variables achieved statistical significance. Higher age ($p<0.001$), participation in relief work ($p<0.01$), and having sustained other physical injury ($p<0.05$) predicted a GHQ score of two or more (Table 9). Thus, age and participation in relief work are good predictors of mental health morbidity as indicated on both regression analyses and the level of statistical significance, and having sustained physical injuries is a weak predictor.

### DISCUSSION

Discussion of Rationale of the study

The scientific study on the mental health aspects of disasters in India is relatively new and yet reasonably strong. The studies reported so far have not covered all types of disaster situations, and many of them lack methodological rigor in terms of appropriate sampling strategy, adequate control group and standardized measures of psychopathology. Central disasters like Marathwada earthquake, Bhopal Gas Tragedy, Orissa Cyclone etc. have attracted attention of mental health researchers and service providers. There are some case study descriptions and a few research studies also. The research studies have focused on psychiatric disorders, sometimes exclusively or predominantly on PTSD, and only a few of them have the rigorousness of research design and method. Some peripheral disasters have also been studied for psychiatric disorders. As described earlier, there have been no systematic research studies on intermediate disaster situations.

This is so inspite of the fact that intermediate disasters occur more frequently than central disasters. Mental health consequences and service needs of central and intermediate types of disasters can be similar as well as different. Therefore, studies of mental health aspects of intermediate disaster is likely to provide useful information for managing mental health aspect of disaster. Further, mental health sequelae of disasters in socially disadvantaged group have not been looked into specifically. The present study is the first of its kind in India which investigated mental health morbidity in under privileged population affected by an intermediate disaster (fire) in a community setting with an experimental design (has a control group).

### Discussion of Methodology of the study

The study employed a "modified cohort" research design wherein the population groups were selected on the basis of exposure to the hypothesized risk factor (fire disaster) viz – the exposed study group and an unexposed control group. Though a truly cohort study would be highly desirable, such a design can be achieved only through rare co-incidence and not by design in disaster epidemiology. In a review of disaster epidemiological studies by Bromet and Dew (1995), only 7 such studies could be located. This review concluded that the vast majority of the disaster studies in international literature have used a 'modified cohort' design in which a representative sample of exposed and non-exposed individuals is selected for study after the exposure occur (Bromet and Dew, 1995). In India only 2 disaster studies have used modified cohort design so far. The ICMR studies on Bhopal Gas Tragedy and on Marathward earthquake (Bhiman, 2001; ICMR, 2000), both of which were central disasters. Therefore the findings of the present study are relevant in general for all the disaster due to its better design and specifically for intermediate disaster situations.

The selection of a comparable control group in a modified cohort design or disaster epidemiology research is a difficult task. The criteria for selection of control group in the present study were quite rigorous as the control group required to be selected from a colony of comparable socio-economic status within the same slum cluster but more than 3 km away from the fire affected colony, having not experienced any fire disaster for past 10 years. After assessing 14 colonies, suitable colony for the control group could be located. Although the best possible efforts were made to identify a comparable residential colony for the control group, in the post havoc analysis for matching the study group and control group some inter-group differences in socio-demographic variables were observed [Table 2(a) & Table 2(b)]. Thus the control group had lower per capita income and more unemployed subjects in comparison to study group, though it had better educational profile. The differences in religion and occupation were due to predominant presence of Muslim population and absence of farmers in study group. Differences though statistically significant, are not indicative of any specific overall difference in profile between the groups since the seemingly related sociodemograhic variables like per capita income, employment status, education, occupational status, were pointing in different directions for both the groups.

Two stage assessments were used to assess for mental health morbidity by using GHQ-12 (Hindi version) and SCAN based interview. By this, we could study mental health morbidity not only in terms of prevalence and pattern of diagnosable psychiatric disorders but also that of psychological ill health and psychological symptoms. GHQ is the most commonly used screening instrument in psychiatric epidemiological studies in general population both internationally as well as in India. It also has been used in disaster epidemiology (McFarlane et al, 1997). Its shorter version GHQ-12 has been validated for use across cultures (Goldberg et al, 1997). Indian adaptation of GHQ including GHQ-12 has been validated in many languages including Hindi and has been widely used by Indian researchers (Shamsunder et al, 1986; Gautam et al, 1987). The cut off point for GHQ-12 has been reported to vary across cultures. In a 15 centre WHO collaborative study on validity of GHQ-12, a score of 6/7 has been found valid as cut off point for Indian population (Goldberg et al, 1997). It was reported that the cut off score of 6/7 was found to be valid for Bangalore centre, with satisfactory validty parameters.

However, we have still used a lower cut off of two so as to maximize the sensitivity of screening instrument in use.

Thus, the present study has many methodological improvements over previous studies on disaster epidemiology in India. The study has modified cohort research design with representative samples of study group and a comparable control group, which only two Indian studies on disaster have so far (ICMR, 2000; Bhiman, 2001) with no Indian studies on intermediate disaster.

It measured psychiatric morbidity in the form of psychiatric disorders on ICD-10 classification system, which were made by a qualified psychiatrist through interview on SCAN based interview format using the Diagnostic Criteria for Research (DCR). In addition to diagnosable psychiatric disorders, general psychological illhealth and some of the common psychological symptoms were also assessed (through GHQ-12) as a part of psychiatric morbidity.

### Discussion of results

### Prevalence of mental health morbidity

The study found a prevalence of psychiatric disorders in the study group two years after the disaster which was comparable to that found after Bhopal gas tragedy (94 per thousand at one and half year post disaster) (Bhiman, 2001). The prevalence in the present study is lower than that found after Marathwada earthquake (ICMR, 2000) which can be expected as the central disasters like severe earthquakes may result in higher psychiatric morbidity. The statistically significant difference between study group and control group on prevalence of psychiatric disorders negates the Null Hypothesis and accepts the Alternative Hypothesis which had been proposed and also corroborates the findings of Indian as well as international studies on disaster epidemiology reviewed earlier in this paper. The findings of this study once again emphasize that intermediate disasters like other disasters lead to increased psychiatric disorders among the affected people even after two years of the disaster. The attribution of causality in such circumstances does have the well-known difficulties and limitations. The findings of the present study bear strength, due to representative sample and the control group from the same urban slum.

The high prevalence of general psychological ill-health in study group than control group as indicated by GHQ-12 again support the international findings in disaster epidemiological research, that a large proportion of mental morbidity in disaster affected population remain below the level of diagnosable psychiatric disorders and indicates the overall psychological ill-health in the affected population. Many of them do suffer from significant distress or disability caused by these subsyndromal psychological symptoms.

However, the prevalence rates of psychiatric disorders are low in both the study group and control group, particularly in the latter as compared to the overall prevalence rates reported in meta-analysis of different epidemiological studies in general population in India (Reddy & Chandrasekhar, 1998). This meta-analysis reported a prevalence rate of 78 per thousand for urban population in India. This lower than expected prevalence rates may either be a true finding or an artifact. The population in our study belonged to the lower socioeconomic status who are subject to repeated stressors of all kinds in their day to day life. This might have increased their psychological immunity. The international literature about the effect of poverty and socio-economic deprivation on psychiatric morbidity is equivocal. While many researchers report higher psychiatric morbidity among people from lower socio-economic strata, Bruce et al (1991) did not find these differences to be statistically significant. In a study on rural children, poverty was only weakly associated with child psychiatric disorders (Costello et al, 2001). Further some researchers found that the association of economic inequalities with higher prevalence of common mental disorders is found only in the affluent populations and not in low income population (Weich et al, 2001). A detailed discussion of this fascinating aspect is beyond the scope of this paper, but the findings of this study support the view that diagnosable psychiatric disorder is lower in the underprivileged communities. Alternatively this might be an artifact. Bromet & Dew (1995) have raised the issue of 'non-response' by the subjects in the study group as well as control group which may lead to underestimation of prevalent psychiatric morbidity due to people's non-response while giving replies during assessment. This still remains a possibility in our study also, though best attempts were made to maintain the interest and involvement of the people in the study by measures like community awareness camps and the provision of treatment or the referral for subjects found to have psychiatric disorders. The second source of artifact is the appropriateness of the overall approach to the assessment of mental health aspects of disasters in developing countries and in cultures like ours. This has been pointed out that universalist or 'etic' approach wherein the singular emphasis is placed on the diagnostic categories of mental disorders usually measured by quantitative techniques may be less appropriate to study mental health aspects of disasters in developing countries, as it is not adequate to understand the full spectrum of behavioural responses to a disaster or trauma. Further, this may lead to over medicalization of social problems as mental disorders (Kleinman and Kleinman, 1985; Patel, 2000; Desai et al, 2002).

### Pattern of Mental Health Morbidity

The most common psychiatric disorders found in the study group were depressive disorders, substance use disorders, generalized anxiety disorder and somatoform disorders. The previous studies in India also has found a similar pattern of psychiatric morbidity. Thus the Marathwada earthquake study reported the depression, anxiety and substance abuse as the commonest disorders while Bhopal gas study reported 94% cases to be "neurotic" (ICMR, 2000; Bhiman, 2001).

It must be noticed that not a single case of PTSD was found at 24 months after the disaster among 1251 subjects affected by the disaster. This indicates the need to avoid the over emphasis and undue focus on PTSD. It is possible that development PTSD as a response to disaster is highly influenced by cultural differences and Indian population may have actually less PTSD cases after the disasters, as compared to the other populations.

The pattern of psychological symptoms in study group suggests that four of five commonest symptoms were suggestive of depression, which indicates that many of these people suffered from subsyndromal depression. Sub-threshold depression and anxiety features have also been reported in Marathwada earthquake affected people (ICMR, 2000). The findings on qualitative research methods in our work on Gujarat earthquake also support these observations (Desai et al, 2002).

These findings emphasize the need of developing culture specific instruments for comprehensive assessment of psychological symptoms in disaster affected populations. They also emphasize the need to prepare the disaster intervention plans to identify and help these people with subsyndromal symptoms who otherwise may not get attention of mental health service providers due to absence of any diagnosable psychiatric disorders.

### Predictors of mental health morbidity

The finding that age and participation in relief work are strong predictors of mental health morbidity are worth some discussions. It is generally well recognized that the elderly persons (over the age of 60 years) are likely to have higher mental health morbidity. This study group provides evidence of higher mental health morbidity with increasing age even in the age range of 16 to 60 years. Qualitative researchers and case study descriptions have indicated higher mental health morbidity and the need for mental health services or psychosocial support in relief workers from outside agencies and also for those workers of the local general population who volunteer for relief work. The statistical finding of "participation in relief work" being a strong predictor in the present study is the scientific validation, possibly the first such evidence, for the increased psychological impact on the relief workers (Desai et al, 2002). The finding about the physical injuries other than burns being a weak predictor of mental health morbidity has important implications and requires further examination. Indeed, the possibility of finding predictors of mental health morbidity need to be explored further to re-examine the findings of this study, since it has very important and pragmatic implications.

### IMPLICATIONS OF THE STUDY

The findings of this study has implications both for mental health service delivery and for research on mental health aspects of disasters.

(a) For Mental Health Service Delivery

- (i) There is a definite need to focus on mental health aspects of intermediate disasters and to plan psychosocial intervention for the affected population as it experiences higher prevalence of mental health morbidity than the unaffected population.
- (ii) Certain population subgroups are at higher risk of developing psychiatric disorders and mental ill-health after disasters. These groups should get priority in the psychosocial intervention programmes of disaster management plan.
- (iii) Meanwhile, there is a need to avoid the possible psychiatricisation of the psychosocial aspects of disaster. Though, many people exposed to disasters do develop psychiatric morbidity, many more exposed similarly remain healthy, possibly due to individually and culturally determined psychoprotective factors. Many also have subsyndromal symptoms which can be attended to by the general health workers and the counsellors (Desai et al, 2002)

(b) For Disaster Research

- (i) The presence of psychological ill-health in many of those who did not have diagnosable psychiatric disorder implies the need for detailed and comprehensive evaluation of psychological symptoms in disaster affected populations. From the limitations of the present study and from our experience in the pilot phase study of the Gujarat earthquake, we recommend that in subsequent studies comprehensive symptom assessment should be carried out, either by using the existing instruments, like the Symptoms Checklist (SCL-90) or preferably by developing new culturally appropriate checklists for disaster situations.
- (ii) There is need to carry out long term longitudinal studies for mental health aspects of intermediate disaster to find out the immediate, short term and long term mental health consequences and the service needs of affected populations.

(iii) There are certain research questions about the mental health aspects of disasters which need further investigation. Thus, the relationship of psychiatric morbidity with socioeconomic status, impact of relief provided to the affected people and the usefulness of predictor variables in either prevention or management of mental health consequences of disasters need further research. Indeed, further work on predictors of mental health morbidity can help significantly in planning and organizing the services.

(iv) The issue of interventions aimed at reducing the mental health morbidity, need to be examined as a logical sequence to the descriptive and analytical epidemiology reported in this paper

### LIMITATIONS OF THE STUDY

Bromet and Dew (1995) have described some of the common limitations of disaster epidemiological studies. These are, emigration out of disaster affected area, exclusion of certain population subgroups from the study, use of convenience samples, recall bias in retrospective inquiries, non-response of study subjects, interviewer bias and confounding factors. Though some of these have been taken care in this study, still there are a few limitations in the present study. Emigration is a constant phenomenon in urban slums as many of the slum dwellers keep on changing their area of residence depending on the availability of job. There is a possibility of differential emigration after the fire disaster i.e. either people affected more severely due to fire or having psychiatric problems are more likely to emigrate to other places or vice versa. We did not assess children in our study though the mental health sequelae of disasters in children are well documented and should have been assessed in this study also. The recall bias was minimized by restricting the psychiatric assessment to the recent part (few weeks) and by defining fire exposure variable in such a way that the chances of recall bias are less. For example, the degree of burn injuries was recorded as mild (if no effect due to injuries occurred on daily activities), moderate (if many daily activities were hampered to the disadvantage of individual and family) or severe (if hospitalized). The interviewer bias due to non-blind status of interviews is another limitation of this study. The use of GHQ-12 for psychological symptoms (in addition to screening) is another limitation as discussed earlier. Lastly some of the factors known to be associated with mental health sequelae, like social support, compensation, and post disaster intervention, could not be assessed in this study.

### CONCLUSIONS

The following conclusions are drawn from the present study:

1. a) There is statistically significant higher mental health morbidity, both in terms of psychiatric disorders and psychological ill health, in the population in an urban slum of Delhi, affected by an intermediate fire disaster, as compared to the control group.

   b) The prevalence of psychiatric disorders is 78.8 per thousand and the prevalence of psychological ill health is 232 per thousand.

2. a) Depression, Substance Use Disorders, Generalized Anxiety Disorder and Somatoform Disorders are the commonest psychiatric disorders in the disaster affected population.

   b) The commonest symptom of psychological ill health are suggestive of depression.

3. Higher age and participation in relief work are good predictors of mental health morbidity and physical injuries (other than burns) is a weak predictor.

Locality of Work: Communities at Bengali Colony and Hathi Basti of Yamuna Pushta slums in Delhi.

Declaration of Interest – This paper is based on ICMR supported research project on “Mental Health Consequences and Service needs of disaster (fire) affected community in an urban slum”

### ACKNOWLEDGEMENTS

1. The Director IHBAS, Faculty and Staff of IHBAS and student volunteers of MA (Psychology) and MA (Social Work) for the initial part of the mental health service delivery carried out. The Director IHBAS and the Ethics Committee of IHBAS for permitting and facilitating the Research Project.
2. The research work reported in this paper is a part of ICMR sponsored research project titled “Mental Health consequences and service needs of a (fire) disaster affected community in an urban slum”. The authors wish to acknowledge the help and facilitation, with scientific advice obtained from Dr. NK Ganguly, Director General, ICMR; Dr. Bela Shah, Sr. DDG, ICMR and the project advisors and the referees to the project proposal.
3. The authors thank all the participants in the study as well as the key informants and community leaders who helped the research team during field work.
4. The assistance provided by the research team members in data collection is acknowledged on record.
5. The authors thank Dr. Rajbir Singh, Dept. of Biostatistics, AIIMS and Mr. Sashi Bhushan Singh and Sushil Kumar of IHBAS for their help in data analysis.

### REFERENCES

Berz, G. (1989) List of major natural disasters, 1960-1987. Earthquake & Volcanoes, 20, 226-228.

Bhiman, A. (2001) Mental Health Consequences of Bhopal gas tragedy. Abstracts of the 17th World Congress of Social Psychiatry, Agra, p.

Bromet, V., Dew, M.A. (1995) Review of psychiatric epidemiologic research on disasters. Epidemiological Review, 17(1), 113-9.

Bruce M.L., Takeuchi D.T., Leaf P.J. (1991) Poverty and psychiatric status. Longitudinal evidence from the New Haven Epidemiological Catchment Area Study. Archives General of Psychiatry, 48, 470-474.

Cobb, S., Lindermann, E. (1943) Neuropsychiatric observations during the Cocoanut Grove fire. Annals of Surgery, 117, 814-824.

Costello E.J., Keeler G.P., Angold A. (2001) Poverty, race / ethnicity and psychiatric disorder: a study of rural children. American Journal of Public Health, 91, 1494-1498.

Desai, N.G., Gupta, D.K., Joshi, P.C. et al (2002) Mental health services needs and service delivery models in the earthquake disaster affected population in Gujarat: A pilot phase study. Report submitted to Indian Council of Medical Research (ICMR).

Gautam, S., Gupta, I.D., Batra, L. et al (1998) Psychiatric morbidity among victims of bomb blast. Indian Journal of Psychiatry 1998, 40, 41-45.

Gautam, S., Nijhawan, M., Kamal, P. (1987) Standardization of Hindi version of Goldberg's General Health Questionnaire. Indian Journal of Psychiatry, 29(1), 63-66.

Goldberg, D.P., Gaten, R., Sartorius, N. et al (1997) The validity of two versions of GHQ in the WHO study of mental illness in general health care. Psychological Medicine, 1997, 27, 191-197.

Green, B.L. (1982) Assessing levels of psychological impairment following disaster. Journal of Nervous and Mental Disease, 170, 544-552.

Gupta D.K., Desai N.G. (2003) Mental Health aspects of an intermediate disaster in an urban slum in Delhi. Executive summary of ICMR Research Project. IHBAS, Delhi

Indian Council of Medical Research (2000) ICMR centre for advanced research on health consequences of earthquake disaster (Marathwada, 1993) with special reference to mental health. Report submitted to ICMR. Pune-Maharastra Institute of Mental Health.

Jacob K.S., Bhugra D, Mann AH (1997) The Validation of the 122 item General Health Questionnaire among ethnic Indian women living in United Kingdom. Psychological Medicine, 25, 1215-17.

Kleinman, A., and Kleinman, J. (1985) Somatization. The interconnection of Chinese society among culture, depressive experiences and the meaning of pain. In.: A Kleinman and B Good (eds), culture and depression, Berkeley: University of California Press 429-490.

McFarlane, A.C., Clayer, J.R., Brobless, C.L. (1997) Psychiatry morbidity following a natural disaster: an Australian bushfire. Social Psychiatry and Psychiatric Epidemiology, 32(5), 261-8.

Narayanan, H.S., Sathyavati, K., Nardev, G., Thakar, S. (1987) Grief reaction among bereaved relatives following a fire disaster in a circus. NIMHANS Journal, 5(1), 13-21.

National Institute of Mental Health and Neurosciences (1997). Proceedings of National Workshops on Psychosocial consequences of disaster held at NIMHANS, Bangalore.

Patel, V. (2000) Culture and the mental health consequences of trauma. The Indian Journal of Social Work, 61(4), 619-631.

Reddy, M.V. Chandrashekhar, C.R., (1998) Prevalence of mental and behavioural disorders in India: A meta-analysis. Indian Journal of Psychiatry, 40(2), 149-157.

Rubonis, A.V., and Bickman, L. (1991) Psychological impairment in the wake of disaster: the disaster - Psychopathology Relationship. Psychological Bulletin, 109(3), 384-399.

Sethi, B.B., Sharma, M., Trivedi, J.K., Singh, H. (1987) Psychiatric morbidity in patients attending clinics in gas affected areas in Bhopal. Indian Journal of Medical Research 1987, 86 (Suppl.) 47-50.

Shamsunder, C., Murthy, S.K., Prakash, N., Subbakrishna, D.K. (1986) Psychiatric morbidity in a general practice in an Indian city. British Medical Journal, 292, 1713-1715.

Sharan, P., Chaudhary, G., Kawathekar, S.A., Saxena, S. (1996) Preliminary report of psychiatric disorders in survivors of a severe earthquake. American Journal of Psychiatry, 15, 556-558.

Sharma, A., Rao, S., Srinivas, S. (1998) Mandi Dabwali fire disaster. Psychological impact on school children after two months - Preliminary findings. Indian Journal of Psychiatry, 40 (Suppl), 51-52.

Shetty, H., Chhabria, A. (1997) Bombay Riots - A case study with an emphasis on psychosocial consequences. Presented at National Workshop Psychosocial Consequences of Disasters help at NIMHANS Bangalore.

Weich, S., Lewis, G., Jenkins, S.P. (2001) Income inequality and the prevalence of common mental disorders in Britain. British Journal of Psychiatry, 178, 222-227.

Weisaeth, L. (1989) A study of behavioural responses to an industrial disaster. Acta Psychiatrica Scandinavica, Suppl, 355, 80, 13-24.

World Health Organisation (1992a) Psychosocial consequences of disasters - Prevention and management. World Health Organisation, Geneva.

World Health Organization (1992b) Schedule for Clinical Assessment in Neuropsychiatry. World Health Organization. Geneva.

World Health Organization (1993) The ICD-10 Classification of Mental and Behavioural Disorders. Diagnostic Criteria for Research. World Health Organization. Geneva.
